# Group Normative Propensities, Societal Positioning, and Childbearing: Ethno‑linguistic Variation in Completed and Desired Fertility in Transitional Central Asia

**DOI:** 10.1007/s11113-022-09701-x

**Published:** 2022-03-11

**Authors:** Victor Agadjanian, Lesia Nedoluzhko

**Affiliations:** 1 Department of Sociology and the International Institute, University of California - Los Angeles, Los Angeles, CA 90095-1551, USA; 2 Independent Researcher, Bishkek, Kyrgyzstan

**Keywords:** Completed fertility, Fertility desires, Ethnicity, Cultural norms, Inequalities, Central Asia

## Abstract

Considerable research in western, low-fertility contexts has examined minority-vs.-majority fertility differentials, typically focusing on minority groups’ cultural idiosyncrasies and on socioeconomic disadvantages associated with minority status. However, the formation and functioning of ethnic complexities outside the western world often diverge from the standard western model and so may their impact on fertility preferences, behavior, and outcomes. We expand on the previous research by analyzing ethnic variation in completed and desired fertility in the multiethnic transitional setting of Kyrgyzstan, where ethnic groups and their ethnolinguistic subparts are characterized by both different stages of the demographic transition and different positioning in the socioeconomic and political hierarchies. Using combined data from two rounds of a nationally representative survey, we find that ethnic-specific levels of completed fertility generally align with culturally shaped group-level normative propensities. In contrast, in desires to have a(nother) child, the ranking of the ethnic segments is more reflective of their collective societal positioning, with more disadvantaged segments having lower fertility desires, regardless of actual number of children and various other characteristics. We also find that ethnic homophily of respondents’ social milieu and their optimism about the future of their ethnic group are positively associated with fertility desires, even though these associations are more potently present among women, compared to men. We relate our findings to the extant scholarship and reflect on their implications for a better understanding of ethno-racial fertility dynamics and differentials in transitional contexts.

## Introduction

Considerable scholarship has examined ethnic and racial variations in fertility behavior and outcomes. The most common initial assumption of this literature is that such variations may reflect ethnoracial differences in individual characteristics, especially those that define socioeconomic status such as educational attainment, employment, income, and wealth. Beyond the ‘characteristics’ perspective, the literature has focused on inter-ethnic cultural differences that may be consequential for the norms and practices that are seen as proximate determinants of fertility such as pre/non-marital sexual partnership, timing of marriage, and use of contraception and abortion (e.g., Agadjanian & Qian, 1997; Caldwell & Caldwell, 1987; Kim & Raley, 2015; Kulu & Hannemann, 2016; Sweeney & Raley, 2014). This ‘cultural’ perspective has been contrasted with the ‘minority status’ perspective, which argues that fertility of ethnic and racial minorities may differ from that of the majority because of characteristics inherent to minority group members’ shared status in societal hierarchy (Goldscheider, 1971; Goldscheider & Uhlenberg, 1969; Kennedy, 1973). This perspective has been further modified and expanded to stress the importance of ethnoracial stratification as a key mechanism shaping opportunity structure and costs of childbearing as well as access to reproductive health services among disadvantaged minorities (Forste & Tienda, 1996; McDaniel, 1996). Additionally, it has been argued that the majority-minority differentials in fertility should be situated within the constantly evolving socio-political context (Greenhalgh, 1990) and socio-demographic environment (Chamie, 1981). Hence, while distinguishing between the cultural and structural dimensions and determinants of fertility, this scholarship has also highlighted their continuous and consequential intersections.

However, most research on ethnoracial variations in fertility has dealt with contemporary western contexts, with a particular focus on native vs. immigrant comparisons, typically attributing higher fertility among immigrants and their descendants to their greater traditionalism/familism, often sustained by barriers to their cultural assimilation and social integration—i.e., a combination of cultural and minority status factors. Yet, ethnic diversity in many non-western settings is fundamentally different as it usually preceded the formation of nation states and corresponding natives-immigrants encounters. While in most such settings one ethnic group may dominate numerically, culturally, and politically, and, in fact, the state may deploy policies aimed at effacing the unique ethnolinguistic and other cultural idiosyncrasies of minority groups, the assimilationist framework, which typically drives the research on fertility of ethnic minorities in the West, is not fully applicable to such settings. At the same time, research on the connections between societal uncertainty and fertility in western settings typically focuses on general labor market and other economic aspects of such uncertainty, without fully addressing possible ethnoracial variations in its nature and levels (e.g., Kreyenfeld, 2010; Sobotka et al., 2011). In this study, we adapt the perspectives generated in the western-focused scholarship on ethnicity and fertility to Kyrgyzstan, a transitional Central Asian nation characterized by considerable ethnic and linguistic diversity, economic yet also political-status inequalities among constituent ethnic groups, and systemic, and occasionally violent, marginalization of ethnic minorities.

## Background

Substantial research in western settings has focused on fertility of minority groups, especially those of non-native origin. Much of the evidence generated by this research points to higher fertility among minorities, compared to majorities, and the literature generally entertains two competing explanations—the cultural explanation, which typically emphasizes greater traditional pronatalism of minority groups, and the minority status explanation, which stresses various aspects of systemic societal disadvantages of minority group members.

A large share of the research on ethnic and racial variations in fertility comes from the United States. Although the majority-minority differentials in completed fertility there have existed continuously, they have been declining recently, and most of the remaining variation is concentrated in the timing of childbearing, the prevalence of non-marital childbearing, contraceptive and abortion use, and the intendedness of births (Guzman et al., 2010; Parrado & Flippen, 2012; Sweeney & Raley, 2014). Yet, the drivers and mechanisms of fertility differentials may vary across minority groups. Thus, Guzzo et al. (2015) found that socioeconomic differentials largely accounted for higher fertility levels among Blacks, compared to non-Hispanic whites, but did not erase the corresponding fertility gap between the latter and Hispanics. The authors suggested that different family values and related union formation practices could explain these residual differences. Westoff and Marshall (2010) linked high fertility among Hispanics to that group’s relatively high religiosity; however, their analysis showed that religiosity does not fully explain the excess of Hispanic fertility. Frank and Heuveline (2005) demonstrated high fertility among Mexican Americans, especially in comparison with declining fertility in Mexico. As these trends challenged the notion of cultural assimilation, the authors proposed to seek explanations for them in the ethnoracially stratified U.S. social context, thus associating high fertility of the Mexican-origin population with its persistent socioeconomic disadvantages. This conclusion aligns with the ethnoracial stratification perspective (Forste & Tienda, 1996; McDaniel, 1996) and parallels the earlier findings by Abma and Krivo (1991) who concluded that limited economic opportunities, rather than cultural characteristics, are a primary driver of elevated fertility among Mexican Americans. Yet, Parrado and Morgan (2008) argued that fertility of descendants of Mexican immigrants converges to the fertility levels of natives, and key fertility determinants, such as education, operate similarly for the two groups, thus essentially asserting the cultural explanation of ethnic fertility patterns and trends.

Beyond the U.S.A., several studies have looked at fertility of immigrants and their descendants in Western Europe. In the United Kingdom, Kulu and Hannemann (2016) found consistently higher fertility among descendants of immigrants, especially of Bangladeshi and Pakistani backgrounds, compared to the native majority, regardless of education or employment. They attributed this pattern mainly to persistent cultural divides. At the same time, some evidence points to a narrowing majority-minority gap in fertility. Thus, in Germany, Krapf and Wolf (2016) documented elevated fertility levels among Turkish immigrants who came to the country as children (i.e., ‘1.5 immigrant generation’), compared to the German majority, but smaller differences for the second-generation immigrants. Dubuc (2012) and Dubuc and Haskey (2010) reported a general reduction of fertility differentials between natives and immigrants, including those from high-fertility countries, across time and generations in the U.K. This research explains the native-vs.-immigrant fertility differentials and trends therein primarily in terms of cultural dynamics of socialization and integration.

Beyond individual-level ethnicity, studies in western settings have also looked at the ethnic community context. Thus, Wilson and Kuha (2018) reported that immigrants’ fertility in the U.K. is lower in less segregated neighborhoods compared to more segregated ones. Abma and Krivo (1991) found a net positive association of the proportion of Mexican Americans in a community with fertility levels among members of that group, which they attributed primarily to economic disadvantages in communities with a high share of Mexican Americans. Yet, their analysis also showed that this share was positively related to the number of recent births even after controlling for available measures of the community economic context. And Lichter et al. (2012), showed that fertility of Hispanic immigrants and their descendants in new settlement destinations in the U.S. is higher than their fertility in traditional destinations, concluding that this pattern is driven by stronger cultural connections among immigrants in new destination communities.

While most studies typically document higher fertility of immigrant minority groups, at least in the earlier stages of their incorporation into the host society, there is also evidence of the opposite direction of the immigrant vs. native fertility divide. Thus, Andersson et al. (2017) showed that fertility of descendants of immigrants in Sweden is generally lower than among individuals of full Swedish roots, even though this difference varies across parity levels. Espenshade and Ye (1994) found in the U.S. that Chinese-American women’s relatively lower fertility reflects that group’s strategy in dealing with discrimination in the labor market: given the steep structural barriers these women faced, reducing fertility was a tool that helped to overcome these barriers. However, as Kennedy (1973) argued using the example of Northern Ireland, this tradeoff mechanism can only work when members of the disadvantaged minority group can see a potential for social mobility.

The scholarship on ethnicity and fertility outside of the western world is much more limited but nonetheless informative. Thus, Poston et al. (2006) looked at the majority-minority fertility differentials in China, noting generally higher fertility levels among minorities. Importantly, however, in the Chinese case, the official one-child policy and the penalties for its violation were strictly applied to the Han majority, while members of minorities were allowed to have two children; this differential application of the policy was a primary factor in the majority-minority fertility differentials. Oyinloye et al. (2017) detected variations in levels of lifetime fertility among different ethnic groups in West Africa, explaining them mainly in terms of ethnic differences in such characteristics as urbanization level and prevalence of polygyny. Other studies in sub-Saharan Africa that documented substantial ethnic variations in fertility levels (e.g., Adebowale, 2019; Kollehlon, 1989, 2003) and fertility desires (Akonor & Biney, 2021) tended to attribute these variations to ethnic cultural norms rather than group minority status.

While focusing on a somewhat different dimension of societal complexity, studies linking fertility to religion also inform our understanding of group-level fertility differentials in non-western contexts. Thus Morgan et al. (2004) in their examination of Muslim vs. non-Muslim fertility in four Asian countries did not find evidence that religion-inspired norms related to women’s status explain higher fertility among Muslims. Sahu et al. (2012) reported higher fertility among religious minorities in India and Bangladesh, attributing it to those minorities’ socioeconomic disadvantages rather than some doctrinal and related cultural characteristics. Notably, in that study, the majority-minority differentials were particularly pronounced in areas of high concentration of minority groups. Similarly, Agadjanian et al. (2009) showed in their analysis of contraceptive use in Nigeria and Tanzania that fertility regulation through contraception tends to be more common in religiously mixed communities, regardless of individual religious affiliation and other factors. These findings parallel those of the earlier cited studies on the connection between ethnic context and fertility in western settings (Abma & Krivo, 1991; Lichter et al., 2012; Wilson & Kuha, 2018). However, importantly, as Chamie (1981) proposed in his analysis of Muslim-Christian fertility differentials in Lebanon, the relative significance of religion-related norms vs. religious groups’ societal status for fertility behavior may vary across different stages of the demographic transition.

Research has also looked into ethnic heterogeneity in fertility levels and patterns in transitional post-Communist multi-ethnic contexts, including Central Asia. While several studies documented fertility differences across ethnic groups, typically explaining them in terms of cultural factors and corresponding stages in the process of the demographic transition (e.g., Denisenko et al., 2012; Nedoluzhko, 2012; Spoorenberg, 2013), some of that research has also highlighted substantial sub-ethnic group differences in childbearing-related practices, which are reflective of the region’s ethnocultural and ethnolinguistic historical trajectories. Thus, in their analysis of induced abortion using the 1995 Kazakhstan Demographic and Health Survey (DHS), Agadjanian and Qian (1997) detected not only a higher likelihood of induced abortion among women of European background compared to members of the titular majority, Kazakhs, but also an instructive variation among the latter: Kazakh women who chose to be interviewed in Russian were more likely to report a pregnancy termination than Kazakh women who chose Kazakh as the language of interview, net of other characteristics. Also in Kazakhstan, Agadjanian, Dommaraju, and Glick (2008) found strong differences between Russian and other European-origin vs. Kazakh women in parity progression, but also significantly higher odds of having the second and third birth among more Russified than less Russified Kazakh women. Both studies attributed these variations to cultural divides between the European-origin population and Kazakhs, but also to the degree of Euro-Russian cultural imprint among the latter. At the same time, the previous research in the region has not directly engaged the minority group status perspective. Our study, therefore, makes an important contribution to this scholarship by incorporating the minority status lens into the analytical narrative.

## Context

The Kyrgyz Republic (Kyrgyzstan) is a country of over 6.5 million people in Central Asia that, became an independent nation after the dissolution of the Soviet Union in 1991. After a sharp decline in the early years following post-Soviet collapse, the country’s economy grew throughout much of the first two decades of this century; yet, with a GNI per capita equivalent to c. 1200 USD, Kyrgyzstan remains one of the poorest Soviet successor states. Like many post-Soviet nations, Kyrgyzstan is a multiethnic country. Ethnic Kyrgyz, the country’s titular group of Turkic origin, form the majority of its population (c. 74%). Kyrgyzstan’s largest ethnic minority, Uzbeks (15%), are also of Turkic linguistic background and are indigenous to the region; Uzbeks reside primarily in the country’s south. Both groups have nomadic roots; however, Uzbeks, unlike Kyrgyz, have long been sedentary. While both groups are Sunni Muslim, Uzbeks are characterized by a generally stronger Muslim identity. Although the two largest groups share Turkic linguistic roots and the Islamic religion, the relations between them have been marked by tensions that occasionally erupted into open confrontations, most recently culminating in massive anti-Uzbek violence in 2010 (Matveeva et al., 2012). Notably, that violence has been said to be at least partially motivated by Kyrgyz-Uzbek economic disparities (Esenaliev & Steiner, 2012). Indeed, Agadjanian and Oh (2020) reported a persistent net earnings advantage of Uzbeks over Kyrgyz. In addition to these main two indigenous groups, Kyrgyzstan has a sizeable Russian-speaking population of European origin and Christian background (mainly ethnic Russians, but also Ukrainians, Belarusians, Germans, etc.) to whom we hereafter also refer summarily as Europeans (c. 7%). Europeans came to Kyrgyzstan as both voluntary and involuntary migrants mainly during the Soviet era. Europeans held considerable political, economic, and cultural clout during the Soviet years, but that clout started to quickly erode after Kyrgyzstan’s independence. Europeans also completed the demographic transition earlier than the two main native groups, which, along with their large-scale post-Soviet emigration from Kyrgyzstan, has led to the shrinking of their population share. Yet, the symbolic importance of their cultural imprint, most prominently represented by the Russian language, has endured, despite the growing efforts by the post-Soviet governments to promote the Kyrgyz language and culture (Agadjanian & Nedoluzhko, 2021). In fact, previous research has identified not only a persistent net earnings advantage of Europeans over Kyrgyz, but also considerable variations within the titular ethnic group based on the degree of its members’ linguistic Russification: the use of the Russian language was associated with higher earnings among ethnic Kyrgyz (e.g., Agadjanian & Oh, 2020). Finally, Kyrgyzstan’s population includes other smaller groups of both indigenous (e.g., Uyghur, Kazakh, Tajik) and non-indigenous (e.g., Tatar, Chechen) roots.

While the ethnic labeling, systematically introduced and enforced in Central Asia in the Soviet era, has remained remarkably rigid and stable after its end (cf. Abramson, 2002), the shifting meanings and positioning of different ethnolinguistic segments in the post-Soviet period have shaped their distinct group-shared identities and sense of belonging, and have translated into different labor market outcomes and diverging levels of societal optimism, political participation, and emigration intentions (e.g., Agadjanian, 2020; Agadjanian & Oh, 2020; Agadjanian, Dommaraju, et al., 2008; Agadjanian, Nedoluzhko, & Kumskov, 2008; Gorina & Agadjanian, 2019). We add to this complex panoply of ethnolinguistic dynamics by analyzing variations in fertility behavior and desires across the ethnic and linguistic divides.

The total fertility rate (TFR) in Kyrgyzstan, which hovered close to four children per women in the late Soviet era, dropped dramatically following the disintegration of the U.S.S.R. and the generalized crisis that it triggered, paralleling trends in other parts of the former Soviet empire (e.g., Agadjanian, Dommaraju, et al., 2008; Agadjanian, Nedoluzhko, & Kumskov, 2008; Frantsuz & Ponarin, 2020; Koehler & Koehler, 2002). After that initial post-Soviet shock, the overall fertility levels stabilized and began to recover after the mid-2000s. According to the Kyrgyzstan DHS conducted in 2012, the country’s TFR was 3.6 children per woman, a slight increase from 3.4 recorded in the previous DHS conducted in 1997 (NSC, 2013: 86). The 2018 Multiple Indicator Cluster Survey (MICS) registered an even higher TFR—3.9 (NSC, 2019: 78). The nationwide estimates based on the National Statistical Committee (NSC) data indicate a more modest increase, with TFR reaching 2.9 in 2009 and 3.3 in 2018 (Denisenko et al., 2012; NSC, 2020). The available data also suggest some variations in early post-Soviet ethnic-specific fertility patterns, although the estimates differ depending on the data source. Thus, according to MICS-based estimates, fertility of Uzbeks, which exceeded that of Kyrgyz in the mid-1990s dropped significantly a decade later (Spoorenberg, 2013: 67). However, the estimates derived from the 1999 and 2009 national censuses produced nearly identical TFR levels for Kyrgyz and Uzbeks—2.9 in 1999 and 3.1 in 2009 (Denisenko et al., 2012). Interestingly, as Nedoluzhko (2012), using data from the 2006 MICS, reported, Kyrgyz were more likely to want another child than were Uzbeks. In comparison, fertility of ethnic Russians, which was already below the replacement level in the early 1990s, showed a considerable increase after the turn of the century, even though specific survey- and census-based estimates differ as in the above case of the two main indigenous groups (see Agadjanian et al., 2013; Denisenko et al., 2012; Spoorenberg, 2013). Although no recent ethnic-specific estimates are available from the NSC, according to MICS 2018 estimates, fertility of Uzbeks was on par with that of Kyrgyz (NSC, 2019: 80) (The MICS report did not estimate ethnic Russians’ TFR because of the small number of that group in the survey sample.) Our nationally representative and relatively recent data, which contain detailed information on reproductive behavior and desires as well as on respondents’ individual ethnic self-identification, their linguistic choices, and their ethnic social context, offer a unique opportunity to test the theoretical perspectives on ethnic variations in fertility in this understudied transitional context.

## Conceptualization and Hypotheses

Our conceptualization engages, juxtaposes, and contrasts the cultural and minority status perspectives. For the cultural perspective, we suggest a holistic approach to the cultural realm and its connection to fertility, which we define in terms of group-level normative propensities. While group normative propensities are multidimensional and situational, the norms and corresponding behaviors that are most consequential for fertility evolve through the process of the demographic transition, of which fertility regulation and limitation are a central part. Collectively shared family and gender norms, marital practices, and related aspirations and expectations are core mechanisms of group cultural reproduction. Importantly, these norms, practices, aspirations, and expectations are produced and reproduced irrespective of individual variations among group members along such axes as urbanity, education, or religiousness.

We adapt the scholarship on minority group status, and especially its ethnoracial stratification lens, to what we define as group societal positioning. This definition is rooted in, but also expands upon, the minority group status concept as it encompasses all ethnic groups and their sub-groups, regardless of the exact nature of their collective identity and of their relative size. Ethnic groups, and their segments, are stratified and hierarchized in any contemporary multi-ethnic society, and group societal positioning is established, reaffirmed, and reassessed in a constantly evolving ethno-economic and ethno-political context reflecting the dynamic allocations of collective economic and political powers. These variations in group positioning on different stratification axes are specific to country’s macro socio-political environment (cf., Greenhalgh, 1990). Importantly, unlike most western contexts of below-replacement fertility, where minority groups are simultaneously disadvantaged on various societal dimensions and where this cumulative disadvantage may perpetuate their relatively high fertility, in many transitional non-western multi-ethnic contexts, an ethnic group’s socioeconomic stature may contrast with its positioning on the political stratification ladder, and these combined yet contradictory structural forces may have a depressing effect on fertility. And again, these group-level status allocations and their fertility implications operate regardless of within-group individual-level diversity in such characteristics as employment, income, or wealth.

While normative propensities and societal positioning are understandably interconnected, they nonetheless capture different aspects of group identity and experiences. We therefore treat them as two competing group-level perspectives in theorizing ethnic variations in reproductive dynamics in a context of above-replacement, and even slightly rising, fertility, where the ethnic makeup is reflective both of cultural diversity and of status inequalities. Unlike most western countries, where comparisons are almost exclusively drawn between the majority and minorities, the ethnoracial hierarchies in Kyrgyzstan, as in many other non-western settings, do not fit neatly into this dichotomy. Specifically, the heterogeneity of the ethnic majority, both in terms of ethnocultural characteristics and social positioning of its subgroups, may be consequential for demographic behavior and outcomes, regardless of other individual-level variations among them. At the same time, in such settings ethnic minorities are often indigenous, rather than immigrant-origin, groups, and share considerable ethnocultural and religious affinity with majorities; in fact, they became ‘minorities’ historically recently, e.g., during the ethno-administrative partition of the former Russian empire following the Soviet takeover. As for outside-origin groups, such as Europeans in Kyrgyzstan, their initial arrival was part of a state-organized or sanctioned population transfer that typically resulted in their superior economic and even ethno-social placement, again, differing greatly from the entry and incorporation trajectories of immigrant-origin groups in the West, where such groups are typically both economically disadvantaged and less advanced in the process of the demographic transition, compared to the host country’s majority.

Our study measures and models the outcomes that are commonly examined in fertility research—completed fertility and desired fertility—and uses them for a better understanding of the ethnic medley and corresponding societal hierarchies in this transitional post-Communist context and of their demographic implications and manifestations. While the two outcomes are closely and causally interconnected, i.e., fertility desires both predict and are predicted by actual fertility (even though the strength of this interconnection is still debated in the demographic scholarship), in this analysis we see them as complementary reflections of group normative propensities and societal positioning. And again, our conceptualization assumes the effects of group normative propensities and societal positioning above and beyond the effects of such universal individual determinants of actual and desired fertility as marital status, education, urbanity, employment, wealth, and religiosity—i.e., the factors that are typically entertained under the ‘characteristics’ perspective.

Our hypotheses for both outcomes of interest focus on ethnic Kyrgyz, and their cultural and status heterogeneity based on the degree of Russification (as proxied by Russian language use), Uzbeks, the largest and relatively economically advantaged yet also most conspicuously vulnerable autochthonous minority, and Europeans, a culturally distinct group of outside origin that has retained some of its Soviet-era economic advantages but, at the same time, has been increasingly, even if indirectly, marginalized in the post-independence political environment. Although we include other ethnic minorities of both indigenous and non-indigenous origin (summarily labeled as Others hereafter) in the analyses, we do not explicitly situate this residual category in our conceptual model because of its small size and ethnic heterogeneity.

In the analysis of *completed fertility*, we look at women’s number of children ever born. From the normative propensity perspective, fertility levels are expected to reflect ethnic cultural norms that impact childbearing: members of culturally more traditional groups would have higher lifetime fertility than members of culturally and demographically less traditional groups. Given the historical association of demographic modernity with the European-origin population and its cultural influence in the region, we hypothesize that ethnic Russians and other Europeans will display lower completed fertility than members of native groups, net of other factors. In the same vein, considering the persistent legacy of the Euro-Russian cultural influence, we also expect that more Russified Kyrgyz will have lower completed fertility than their less Russified co-ethnics. Finally, the normative propensity perspective suggests that fertility levels of less Russified Kyrgyz and Uzbeks, the two most traditional segments of society in the conventional demographic-cultural sense, will be similar.

The societal positioning perspective, as formulated above, suggests alternative hypotheses. Thus, more Russified Kyrgyz, who combine the advantage of titular ethnicity with practical benefits of Russian language use, will have higher completed fertility than their less Russified co-ethnics. At the same time, members of the directly threatened, even if economically advantaged group, i.e., Uzbeks, will have lower completed fertility than members of the other groups. Finally, the societal position of Europeans, which blends the residual economic advantages with gradual, even if subtle, societal marginalization, suggests that their fertility will be closer to that of less Russified Kyrgyz.

For the analysis of *fertility desires*, we consider both the overall desire to have a(nother) child and the desired timing—within two years or later—of the next birth. At the individual level, our hypotheses echo those proposed for completed fertility. Thus, the normative propensity perspective suggests ethno-linguistic distinctions in fertility desires that would be comparable to those in the number of children ever born: net of other factors (including the number of living children), European ethnicity will be associated with lower desire to have a child, regardless of the time horizon. The cultural Russification premise is also extended to hypothesize the variation in fertility desires among the titular group: more Russified Kyrgyz are expected to be less likely to want a child than their less Russified counterparts, and this difference should not be affected by preferred timing of future fertility. At the same time, the normative propensity perspective, as in the cases of completed fertility, suggests no difference between less Russified Kyrgyz and Uzbeks in desired fertility.

Again, as in the case of completed fertility, the group societal positioning lens entails an alternative set of hypotheses. Specifically, if a group’s position in the societal hierarchy is positively associated with pronatalism, then more Russified Kyrgyz, the de facto privileged segment of society, should be more likely to desire another child than the other segments, including their less Russified co-ethnics, and this difference should be particularly pronounced in longer-term fertility desires. In comparison, ethnic Uzbeks, the minority group of residually high socioeconomic status that has been a target of systematic marginalization and direct violence, are hypothesized to be least likely to want a child. Moreover, given the relatively recent shock of ethnic violence that this group experienced, this tendency will be especially noticeable in shorter-term fertility desires. And finally, Europeans’ continuing economic advantages yet also gradual status decline should situate their fertility desires between those of Uzbeks and less-Russified Kyrgyz, net of other individual characteristics.

In the analyses of desired fertility, we also look beyond the individual-level markers of ethnic identity. At what we consider the meso-level of ethnic belonging, here represented by the ethnic makeup of individuals’ non-family social milieu, we hypothesize that having friends/close acquaintances of same ethnicity will be associated with a higher likelihood of wanting a(nother) child, either sooner or later, compared to having an ethnically diverse close social circle, after individual ethnic self-identification as well as other relevant characteristics are controlled. Finally, at what we define as the macro-level ethnic comfort, we look at individuals’ expectations for the general situation of their ethnic group in the near future. We hypothesize that regardless of ethnic self-identification and other factors, greater optimism about the near-future prospects of own ethnic group will be associated with increased fertility desires, net of other factors. We also expect that the positive effect of such optimism will manifest itself most strongly with respect to shorter-term fertility desires.

Following the dominant tradition of fertility research, the analyses of desired fertility focus primarily on women. However, we also conduct complementary analyses for men. Men’s fertility attitudes and desires may differ from women’s, reflecting different levels of gendered power, nature and scale of labor force participation, as well as established cultural norms and expectations (e.g., Bankole & Singh, 1998; Doepke & Tertilt, 2018; Miettinen et al., 2011; Shreffler et al., 2010; Thomson, 1997). We also acknowledge that the meaning of ethnic identity and belonging may differ by gender, which, in turn, may potentially affect the hypothesized associations. While our general expectations and specific hypotheses are gender-neutral, our gender-specific explorations may shed important light on these nuances.

## Data and Methods

We use combined data from two rounds of the Social, Economic, and Migration Processes in Kyrgyzstan (SEMPK) survey, a nationally representative household survey conducted in 2011 (round I) and 2017 (round II). Both rounds used a multistage cluster design and took place in the same communities (villages or city boroughs), selected at the time of the first round with a probability proportional to size. In those communities, households were randomly selected in each round, and in each selected household one randomly chosen resident, woman or man, aged 18–49 was interviewed. In each round, the sample size was 2030, and no individuals were interviewed in both rounds. The survey questionnaires, which were nearly identical in the two rounds, were administered face-to-face in the language of respondent’s choice and contained a variety of questions, including those on ethnicity, language proficiency and use, number of everborn and surviving children, and long- and short-term fertility desires. The survey design and the questionnaire content were approved by the Institutional Review Boards of Arizona State University and the University of Kansas (USA).

Our first outcome, women’s completed fertility, is the total number of children a woman aged between 18 and 49 has had by the time of the survey, regardless of the survival status of her children. The second outcome is whether a respondent, woman or man, wants to have more children anytime in the future. It is operationalized as a dichotomy—wants to have a(nother) child vs. does not want to have a child. The third, and last, outcome of interest is a refinement of the second one: it breaks down individuals who want to have a child in the future into those who would like to have a child within the next two years and those who would like to have a child later. The fertility desires analyses exclude female respondents who considered themselves infecund. They also exclude female and male respondents who did not know or were unsure whether they wanted to have another child. We acknowledge potential heterogeneity of the subsample that provided “don’t know/unsure” responses as such responses may reflect a fairly wide spectrum of fertility preferences (cf., Becker & Sutradhar, 2007). However, given our conceptual focus on assessing the implications of group-level comfort and optimism, we believe that the chosen operationalization of desired fertility is best suited for this theoretical purpose.

Based on the survey responses, we classify ethnic self-identification into four categories—Kyrgyz, Uzbek, European (including ethnic Russian, Ukrainian, German, etc.), and Other. Notably, although respondents could choose multiple ethnicities, almost all of them picked only one. Next, following our conceptual interest and guided by prior research (e.g., Agadjanian & Dommaraju, 2011; Agadjanian & Oh, 2020; Nedoluzhko & Agadjanian, 2010), we subdivide ethnic Kyrgyz into those who reported using only or mainly the Russian language outside the home (whom we label ‘more-Russified’) and those who reported using only/mainly the Kyrgyz language (‘less-Russified’). To test our hypothesis regarding the effect of the ethnic composition of one’s social milieu, we use responses to the survey question that asked participants to identify the ethnicity of their friends/close acquaintances who live in the same community but not with them. Based on the responses, we operationalize this variable as a dichotomy: all friends/acquaintances are of the same ethnicity as respondent’s vs. of other/different ethnic backgrounds. Finally, for what we defined as short-term macro-level ethnic optimism, we formulate a dichotomous variable: the respondent expects the situation of own ethnic group to improve in the near future vs. expects it to worsen/remain the same or is unsure.

We first fit a set of two Poisson regression models predicting the number of children ever born. These analyses include all female respondents aged 18–49 (the combined two-round analytic sample, which excludes cases with missing values on the outcome or any covariates, is N = 2122). In the first model, ethnic groups are compared. The second model accounts for the degree of linguistic Russification among Kyrgyz. For the analysis of desired fertility, we fit separate models for women (aged 18–45, N = 1597) and men (aged 18–49, N = 1714). For each gender, we start with a pair of binomial logistic regression models predicting the desire to have a(nother) child any time in the future. Then, we fit a pair of multinomial logistic regression models with the following outcome: wants a child within the next two years, wants a child later, and does not want a child (reference). As we do for the two previous outcomes, we fit both the ethnicity-only and ethno-linguistic (i.e., separating more-Russified and less-Russified Kyrgyz) models. All the desired fertility models include ethnic homophily of personal network and ethnic macro-optimism as predictors.

All models control for respondent’s age; the fertility desire model also includes age squared to account for possible non-linearity in the association of age with desired fertility. All models control for educational level (general secondary or less; secondary special; at least some tertiary), current marital status (married vs. not), and marital history (married before vs. not). We do not control for marital partner’s ethnicity (for respondents currently in marital partnerships) because the overwhelming share of marital partnerships were of same ethnicity. The models also control for respondent’s current employment status operationalized into four categories: not employed; self-employed; employed in the state sector; employed in a private company/organization. Household’s socioeconomic standing is defined based on the household monthly income and is operationalized into four categories: low tier; medium tier; high tier; and unknown/unreported. All models also control for respondent’s self-assessed religiosity (very religious vs. not) and area of residence (rural vs. urban). Survey round (I or II) is also included as a control in all models: although the two rounds had the same sampling design, used nearly identical instruments, and were separated by only six years, the timing of each round and the related socio-economic, political, and ethno-cultural context could have potential implications for the outcomes of interest. The fertility desire models also include a control for the number of living children.

We acknowledge that some of the covariates in the completed fertility model may not be fully exogenous to the outcome. We also acknowledge that men may under-report the number of their children, but we have no reason to expect any systematic variation across ethno-linguistic boundaries in such underreporting. We do not match women’s desires with those of their partners, and vice versa, because partner’s fertility desires are only available from the respondents’ reports and because considering partner’s desires would exclude currently unpartnered individuals from the analysis. All multivariable models account for the survey cluster design and are fitted in Stata.

## Results

### Descriptive Results

Table 1 presents the descriptive results for women’s completed fertility. It shows distinctively lower fertility among European women. Uzbeks’ fertility is similar to that of Kyrgyz as a whole. However, the two subgroups of the titular ethnicity display a notable contrast, with less-Russified Kyrgyz having distinctively higher completed fertility than their more-Russified co-ethnics. The descriptive results for desired fertility are presented in Table 2. Of the four groups of interest, Uzbeks are least likely to want a(nother) child anytime in the future. On the other end of the spectrum are more-Russified Kyrgyz, with less-Russified Kyrgyz situated between their more-Russified co-ethnics and Uzbeks. Europeans’ fertility desires are generally close to those of less-Russified Kyrgyz. The table also shows considerable cross- and within-group variations in desired timing of next birth. Thus, less-Russified Kyrgyz have a substantially higher percentage of those who want a child sooner than later, while the corresponding differences among their more-Russified co-ethnics is much smaller. The desired timing pattern of Uzbeks resembles that of less-Russified Kyrgyz even though it is less pronounced. Finally, the share of Europeans wanting a child sooner is slightly higher than the share of those wanting it later. Overall and across all the ethnolinguistic subgroups, men have a higher proportion of those who want a(nother) child than do women, conforming to the nearly universal global pattern (e.g., Doepke & Tertilt, 2018), but the ethno-linguistic variations within each gender are generally similar.

### Multivariable Results

Table 3 shows the results of the Poisson regression models predicting the number of children ever born to women aged 18–49. The first section shows the results of the model that treats members of the titular ethnicity as a single group. These results strongly align with the normative propensity perspective: regardless of other factors, European-background women have distinctively lower completed fertility, compared to women of the native groups. At the same time, no visible difference among the native groups is noticeable. The second model disaggregates the titular group by the degree of Russification. It shows a strong statistical contrast between more-Russified and less-Russified Kyrgyz, the latter having significantly higher completed fertility, net of other characteristics. Uzbeks are not statistically different from less-Russified Kyrgyz. At the same time, while more-Russified Kyrgyz are closer to Europeans, the difference between the two groups is pronounced and statistically significant (not shown). Overall, the ranking of the ethno-linguistic groups provides support for the normative propensity perspective, rather than the societal positioning perspective.

Next, we fit the models that predict fertility desires among women and men. These models, in addition to testing for variations across ethnic self-identification, also address the role of what we defined as meso- and macro-level ethnic belonging dimensions. The results of the gendered models predicting the desire to have a(nother) child at any time in the future are shown in Table 4. In the women’s model that includes ethnic Kyrgyz as a single group (4.A.1), this group displays a significantly higher likelihood of wanting a child than any of the other groups included in the comparison. The difference is particularly pronounced with Uzbek women, arguably the most culturally traditional segment of the sample. The difference between Uzbek and European women is not statistically significant (not shown). The corresponding results for men (4.B.1) are generally similar, with both European and Uzbek men being less likely to want a child, compared to Kyrgyz. When the titular group is disaggregated by the degree of linguistic Russification (4.A.2 and 4.B.2), more-Russified Kyrgyz women are significantly more likely to want a child than their less-Russified co-ethnics. The intra-Kyrgyz difference among men points in the same direction, but it is not statistically significant. These results generally contradict the normative propensity perspective and instead conform (at least in the women’s case) to the hypothesis based on the societal positioning argument. Likewise, the persistently low fertility desires of Uzbeks support the societal positioning perspective, i.e., that membership in a directly threatened group of residually high socioeconomic status will be negatively associated with fertility desires. Notably, the Uzbek distinction is strongly present in the women’s and men’s models alike. The contrast between less-Russified Kyrgyz and Europeans, suggesting greater pronatalism among the former, is also statistically significant in both gendered models even though it appears more pronounced among men. This contrast is more compatible with our hypothesis based on the normative propensity perspective, although the European pattern also fits with what could be expected for a group with declining social positioning.

When we look at the effects of meso- and macro-level ethnic belonging markers, we find that women who have friends/close acquaintances of only their own ethnicity are more likely to want a child than women with ethnically mixed friendships, regardless of individual ethnic self-identification and other factors. Similarly, with respect to group macro-level prospects, women who expect the situation of their ethnic group to improve in the next few years are more likely to want a child. These results support our hypotheses. In comparison, among men, the effects of the two predictors point in the same direction, yet they are not significant for the ethnic composition of friends/acquaintances and marginally significant (*p* = 0.07) for the assessment of own group’s prospects.

Table 5 shows the results of the multinomial logit model predicting the desired timing of future fertility—within the next two years or later (with not wanting a child as the reference category). Most conspicuously, the results further support the societal position perspective for the most vulnerable group: ethnic Uzbek, men and women alike, are significantly less likely to want a child either sooner or later (with no anticipated differences between short- vs. long-term desires). In comparison, the results show that Europeans’ relative antinatalism, which, as we argued, is primarily reflective of normative propensities, is concentrated mainly in shorter-term fertility intentions. In contrast, the internal Kyrgyz divide, which we attributed to more privileged status of more-Russified Kyrgyz under the societal positioning perspective, is fully manifested in longer-term fertility intentions and is statistically significant at the conventional level among women only (in the men’s models it is marginally significant, at *p* = 0.07).

At the meso-level of ethnic belonging, the results show that having friends/acquaintances of same ethnicity positively affects longer-term desire to have a child, relative to wanting no more children, and this effect is, again, statistically significant among women only (for shorter-term desires the effect is marginally significant among women). Finally, as we expected, the anticipation of the improvement of own ethnic group’s situation in the next few years is positively associated with shorter-term fertility desires. This association is fully statistically significant for women, but it also approaches the conventional threshold of significance in the men’s model (*p* = 0.06).

The effects of some other covariates are also noteworthy. Not surprisingly, being currently married and having been in marriage before both have a positive association with completed fertility. In comparison, for fertility desires, being currently married has a positive effect while previous marital experience has a negative effect; however, both effects are statistically significant only among women. Education only marginally affects women’s completed fertility and has no association with fertility desires. Employment status generally is not related to either outcome among women but shows some statistically significant relevance to men’s fertility desires. In comparison, household income is negatively associated with completed fertility but displays no association with fertility desires for either gender. Rural residents have higher completed fertility than urban dwellers, although this difference is statistically significant only in the model where Kyrgyz are treated as a single group. Interestingly, rural residence is negatively associated with fertility desires, but it is so for men only. High religiosity is a strong predictor of women’s completed fertility; however, surprisingly, it shows no net association with fertility desires. Finally, the models display an increase in both completed fertility and fertility desires in the second round of the survey, compared to the first round conducted six years earlier. While these patterns generally fit with the longer-term trends in fertility in Kyrgyzstan available from other sources (Denisenko et al., 2012; NSC, 2020) and may reflect the gradual stabilization in the country’s economic situation and socio-political climate in the between-round period, exploratory analyses suggest that the rise, while modest overall, was more pronounced among Europeans than among other groups, again, aligning with the earlier post-Soviet tendencies (Agadjanian et al., 2013; Spoorenberg, 2013). (The results of the exploratory tests are not shown but available upon request.) These temporal patterns as well as the variations detected on other covariates, require separate investigations.

## Discussion and Conclusion

In this study, we projected the extant scholarship on ethnicity and fertility, which typically contrasts the demographically more advanced and economically privileged majorities with demographically more traditional and economically disadvantaged minorities in western post-transitional contexts (e.g., Frank & Heuveline, 2005; Kulu & Hannemann, 2016; Parrado & Morgan, 2008; Sweeney & Raley, 2014), onto a multi-ethnic society where ethnic and linguistic lines reflect historico-cultural legacies, ongoing experiences of the demographic transition, and evolving social hierarchies. In doing so, we identified and tested two interrelated yet distinct theoretical perspectives defining group-level historical experiences and decisional repertoires—normative propensity vs. societal positioning.

The detected variations in completed fertility corresponded clearly to the normative propensity perspective, as Europeans, the group historically most advanced in the process of fertility transition, had by far the smallest number of children ever born, followed, at a statistically significant distance, by more-Russified Kyrgyz, the segment of the titular majority that has been greatly impacted by the Euro-Russian cultural influence. The less-Russified Kyrgyz and members of the Uzbek minority had the highest numbers of children among the examined groups, regardless of other factors.

The desired fertility analysis produced a rather different ethno-linguistic lineup. Most conspicuously, Uzbeks, the ethnic minority that could be expected to be highly ‘pronatalist’ if judged through the prism of conventional demographic transition reasoning rooted in the degree of cultural traditionalism, displayed remarkably weak desires for additional children, even after controlling for the number of living children and other potential individual-level determinants of desired fertility. Notably, this pattern, at least with respect to the contrast between Uzbeks and Kyrgyz, echoes that reported by Nedoluzhko (2012) for the mid-2000s, i.e., well before the eruption of massive anti-Uzbek violence in 2010, suggesting that the depression of Uzbeks’ fertility desires was not triggered by those tragic events and instead reflected the gradual and continuous marginalization of this once economically advantaged group. At the same time, while low desired fertility of Europeans generally aligns with both the normative propensity and social positioning perspectives, the standing of Europeans in the hierarchy of desired fertility did not set them apart from Uzbeks, contrary to the case of completed fertility: in fact, apparent antinatalism of Uzbek women is stronger than that of Europeans in statistical terms, especially when it comes to longer-term fertility desires. Finally, among Kyrgyz women, those more-Russified, who had lower completed fertility than their less-Russified co-ethnics, were, at the same time, distinctly more pronatalist, compared to them, irrespective of the number of living children and other characteristics; notably this intra-Kyrgyz gap was fully significant with regard to longer-term fertility desires. This pattern contradicts the normative propensity premise and instead fits with the notion that advantageous societal positioning, which in the case of more-Russified Kyrgyz is shaped by a combination of privileges associated with the titular status (de jure privilege) and Russian-language usage (de facto privilege), may boost fertility desires. In sum, these findings add important nuances to those from western contexts where successful cultural and socioeconomic integration of ethnic minorities is typically assumed to reduce their fertility, and accordingly, the persistently higher fertility among minorities is seen as a sign of failure of their integration (e.g., Andersson et al., 2017; Dubuc, 2012; Espenshade & Ye, 1994; Frank & Heuveline, 2005; Guzzo et al., 2015; Kulu & Hannemann, 2016; Parrado & Morgan, 2008).

Although our data do not allow us to further investigate the divergence in ethnic-specific patterns of completed vs. desired fertility, some tentative explanations can be proposed. Thus, completed fertility reflects cumulative experiences, including those of a relatively distant past, whereas fertility desires and corresponding future expectations and preferences, while impacted by those experiences, are more influenced by current circumstances. In addition, fertility desires may reflect ethnic group members’ more general confidence and optimism, which, in turn, are likely to be influenced by both perceived and experienced vulnerabilities associated with ethnic group membership (cf. Agadjanian, 2020). Although much of demographers’ attention has understandably focused on correspondence between fertility desires and subsequent fertility behavior (e.g., Bongaarts & Casterline, 2018; Quesnel-Vallée & Morgan, 2003; Schoen et al., 1999; Westoff & Ryder, 1977), we caution against a narrow vision of desired fertility as a mere predictor, accurate or not, of actual fertility performance. Instead, we view it as a complex interactive and evolving product of cognitive schemas and social environments (see Bachrach & Morgan, 2013; Bhrolcháin & Beaujouan, 2019; Hayford, 2009; Iacovou & Tavares, 2011; Trinitapoli & Yeatman, 2018) that reflects individuals’ broader constraints, aspirations and expectations, irrespective of subsequent pregnancies and births. Finally, and no less importantly, we should again acknowledge that in real life the cultural-normative and socio-structural forces, and their corresponding mechanisms, while different in origin, operate in dynamic interconnection. In fact, persistent status differentials are gradually internalized as culturally normative, while cultural norms are infused into the construction of social boundaries.

Our findings also help to expand the research on ethnicity and fertility beyond individual markers of ethnic identity. Thus, regardless of specific group membership and other individual-level factors, the ethnic homophily of women’s personal networks is associated with greater pronatalism, and this pattern is particularly strong with respect to longer-term fertility desires. With appropriate caveats, this finding resonates with those of studies that examined the effects of community ethnic or religious homogeneity on individual fertility behaviors and outcomes (e.g., Abma & Krivo, 1991; Agadjanian, Dommaraju, & Glick, 2008; Lichter et al., 2012; Sahu et al., 2012; Wilson & Kuha, 2018). Also among women, the expectation of an improvement in own ethnic group’s situation in the country in the near future had a net positive association with desired fertility and, not surprisingly, this association was particularly pronounced and statistically significant for shorter-term fertility desires. In comparison, among men, these associations, while pointing in the same direction, were not significant for the effects of ethnic network homophily and only marginally significant for the effects of perception of group’s short-term societal prospects. These differences may be tentatively interpreted as indicating greater importance of the social context, especially of the more immediate social milieu, for women’s views and aspirations around childbearing and parenting, compared to men’s.

We acknowledge several limitations of our study. Thus, the cross-sectional nature of the data constrains causal inferences, especially with respect to earlier fertility behavior and its proximate determinants, such as premarital sexual partnership, frequency of sexual intercourse, or use of contraceptive and abortion services, which may account for some of the observed patterns (cf., Agadjanian & Qian, 1997; Agadjanian, Dommaraju, & Glick, 2008; Dommaraju & Agadjanian, 2008). Importantly, however, fertility options and choices in Kyrgyzstan have never been subject to any explicit or even implicit ethnically determined governmental management as was, for example, the case of neighboring China (Poston et al., 2006). The lack of information on the evolution of personal network composition over time prevents us from assessing the effect of local ethnic context on group members’ fertility trajectories. We cannot account for continuing emigration of Europeans and its selectivity, which may, at least to some extent, be linked to the relatively pronounced temporal variations among this segment detected in exploratory analyses. Overall, the relatively small sizes of the ethnic minority subsamples do not allow for generating reliable estimates of ethnic-specific TFRs. Also, as in any survey data analysis, we acknowledge that our ethnolinguistic definitions cannot fully capture the dynamic nuances of group identity and boundary formation and functioning (cf. Nandi & Platt, 2015; Wimmer, 2013). Yet, we note again, that in this post-Soviet setting, the ethnic self-labelling remains generally very rigid and unconditional, especially because inter-ethnic marital mixing is still quite uncommon. We, of course, also acknowledge that in this rapidly changing societal context, the meaning and expressions of these patterns and boundaries will inevitably transform, and these transformations and their fertility implications will need specialized investigations using data over larger time spans that our two-round survey.

These limitations notwithstanding, our findings usefully contribute to a better understanding of the association between ethnicity and fertility in transitional multi-ethnic settings. Our findings can also inform policies and actions in the areas of family and fertility: while such policies and actions are unlikely to focus explicitly on specific ethnic or ethno-linguistic segments of society, they should heed and reflect group-level cultural propensities and structural vulnerabilities, in addition to, and in conjunction with, other factors shaping reproductive and family outcomes.

## Figures and Tables

**Table 1:** Number of children ever born to women by ethnicity/ethno-language (mean)

Ethnicity/ethno-language	Women (aged 18–49)
Kyrgyz all	2.19
Less-Russified	2.52
More-Russified	1.43
Uzbek	2.19
European	1.34
Other	2.18
All	2.12
*N*	2122

**Table 2:** Desired fertility by ethnicity/ethno-language and gender (percent)

Ethnicity/ethno-language	Women (aged 18–45)	Men (aged 18–49)
*Wants a(nother) child, anytime*		
Kyrgyz all	77.18	83.69
Less-Russified	73.23	79.98
More-Russified	85.56	92.02
Uzbek	62.31	72.45
European	66.67	80.27
Other	63.64	85.37
*Wants a(nother) child, within two years*		
Kyrgyz all	46.08	53.36
Less-Russified	48.43	54.15
More-Russified	41.11	51.60
Uzbek	37.69	46.04
European	36.23	41.50
Other	40.26	56.10
*Wants a(nother) child, later*		
Kyrgyz all	31.11	30.33
Less-Russified	24.80	25.83
More-Russified	44.44	40.43
Uzbek	24.62	26.42
European	30.43	38.78
Other	23.38	29.27
*N*	1597	1714

**Table 3:** Poisson regression model predicting the number of children ever born, women aged 18–49, coefficients (t-statistics in parentheses)

*Predictors and controls*	1	2
Ethno-linguistic background		
Kyrgyz all (ref.)	0	-
Less-Russified (ref.)	-	0
More-Russified	-	−0.16 (−3.46)^**^
Uzbek	−0.04 (−0.96)	−0.07 (−1.58)
European	−0.38 (−6.67)^**^	−0.43 (−7.30)^**^
Other	0.04 (0.63)	0.01 (0.10)
Age	0.04 (26.38)^**^	0.04 (26.84)^**^
Currently married [not married]	1.08 (18.11)^**^	1.07 (17.70)^**^
Previously married [not previously married]	0.32 (6.56)^**^	0.32 (6.51)^**^
Education [basic and secondary general]		
secondary special	−0.04 (−1.16)	−0.02 (−0.72)
some tertiary or higher	−0.06 (−1.72)^+^	−0.03 (−0.81)
Very religious [somewhat or not religious]	0.11 (2.44)^*^	0.11 (2.38)^*^
Employment status [not employed]		
employed in state sector	0.00 (−0.11)	−0.01 (−0.22)
employed in private company	−0.02 (−0.43)	0.00 (−0.01)
family business or self-employed	−0.03 (−0.79)	−0.03 (−0.75)
Household income per person, tier [low]		
medium	−0.11 (−3.10)^**^	−0.10 (−2.99)^**^
high	−0.39 (−10.00)^**^	−0.37 (−9.42)^**^
not known/not reported	−0.19 (−2.77)^**^	−0.17 (−2.52)^*^
Lives in rural area [lives in urban area]	0.08 (2.27)^*^	0.05 (1.63)
Survey Round II [Round I]	0.08 (2.81)^**^	0.08 (2.78)^**^
Intercept	−1.62 (−19.97)^**^	−1.57 (−19.59)^**^
*N*	2122	

Notes: Reference categories in brackets. Significance level:

+ p<0.10

* p<0.05

** p<0.01.

**Table 4:** Binomial logistic regression models predicting the desire to have a(nother) child, women (aged 18–45) and men (aged 18–49), coefficients (t-statistics in parentheses)

*Predictors and controls*	A. Women		B. Men	
	A.1	A.2	B.1	B.2
Ethno-linguistic background				
Kyrgyz all (ref.)	0	-	0	-
Less-Russified (ref.)	-	0	-	0
More-Russified	-	0.64 (2.10)^*^	-	0.40 (1.28)
Uzbek	−1.13 (−4.17)^**^	−0.99 (−3.63)^**^	−1.08 (−4.22)^**^	−0.96 (−3.53)^**^
European	−0.87 (−3.56)^**^	−0.62 (−2.41)^*^	−1.12 (−3.58)^**^	−0.96 (−2.73)^**^
Other	−0.99 (−2.47)^*^	−0.83 (−2.03)^*^	0.25 (0.44)	0.38 (0.67)
All friends are of same ethnicity [not all]	0.39 (2.13)^*^	0.41 (2.22)^*^	0.25 (1.08)	0.28 (1.23)
Thinks that situation of own ethnicity will improve in near future [does not think so]	0.32 (2.02)^*^	0.35 (2.24)^*^	0.35 (1.75)^+^	0.37 (1.86)^+^
Age	0.45 (3.97)^**^	0.45 (3.90)^**^	0.18(1.40)	0.18 (1.38)
Age^2^	−0.01 (−5.36)^**^	−0.01 (−5.28)^**^	−0.00 (−2.79)^**^	−0.00 (−2.76)^**^
Currently married [not married]	1.26 (4.48)^**^	1.30 (4.58)^**^	0.53 (1.39)	0.50 (1.31)
Previously married [not previously married]	−0.82 (−3.34)^**^	−0.86 (−3.57)^**^	0.21 (0.68)	0.21 (0.66)
Number of living children	−0.85 (−9.55)^**^	−0.85 (−9.44)^**^	−0.61 (−7.18)^**^	−0.60 (−7.13)^**^
Excellent/good self-rated health [not excellent/good]	0.29 (1.41)	0.31 (1.47)	0.06 (0.28)	0.06 (0.26)
Education [basic and secondary general]				
secondary special	0.26 (1.04)	0.20 (0.80)	−0.23 (−0.94)	−0.24 (−0.98)
some tertiary or higher	0.27 (1.09)	0.15 (0.61)	0.05 (0.17)	0.01 (0.03)
Very religious [somewhat or not religious]	0.08 (0.24)	0.09 (0.28)	0.08 (0.28)	0.10 (0.34)
Employment status [not employed]				
employed in state sector	−0.03 (−0.13)	−0.01 (−0.05)	0.77 (2.84)^**^	0.76 (2.75)^**^
employed in private company	0.20 (0.74)	0.15 (0.53)	0.47 (1.32)	0.41 (1.14)
family business or self-employed	0.10 (0.53)	0.10 (0.48)	0.57 (1.96)^+^	0.58 (1.96)^+^
Household income per person, tier [low]				
medium	−0.23 (−1.11)	−0.26 (−1.30)	0.14 (0.62)	0.09 (0.41)
high	−0.19 (−0.74)	−0.25 (−0.96)	0.29 (1.07)	0.25 (0.90)
not known/not reported	0.17 (0.54)	0.10 (0.34)	0.79 (1.67)	0.76 (1.60)
Lives in rural area [lives in urban area]	0.10 (0.50)	0.19 (0.99)	−0.61 (−2.55)^*^	−0.54 (−2.21)^*^
Survey Round II [Round I]	0.74 (3.21)^**^	0.77 (3.32)^**^	0.73 (3.27)^**^	0.72 (3.24)^**^
Intercept	−2.90 (−1.55)	−3.03 (−1.59)	2.01 (0.91)	1.90 (0.86)
*N*	1597		1714	

Notes: Reference categories in brackets. Significance level:

+ p<0.10

* p<0.05

** p<0.01.

**Table 5:** Multinomial logistic regression models: desired timing of future fertility: never (reference) vs. within two years or later, women (aged 18–45) and men (aged 18–49), coefficients (t-statistics in parentheses)

*Predictors and controls*	A. Women		B. Men	
	A.1	A.2	B.1	B.2
*Wants child within two years*				
Ethno-linguistic background				
Kyrgyz all (ref.)	0	-	0	-
Less-Russified (ref.)	-	0	-	0
More-Russified	-	0.41 (1.17)	-	0.32 (0.97)
Uzbek	−1.16 (−4.16)^**^	−1.08 (−3.84)^**^	−1.10 (−4.09)^**^	−1.01 (−3.49)^**^
European	−1.04 (−3.83)^**^	−0.89 (−3.06)^**^	−1.37 (−4.17)^**^	−1.25 (−3.39)^**^
Other	−0.98 (−2.40)^*^	−0.90 (−2.14)^*^	0.28 (0.46)	0.38 (0.62)
All friends are of same ethnicity [not all]	0.36 (1.85)+	0.37 (1.87)+	0.23 (1.00)	0.27 (1.11)
Thinks that situation of own ethnicity will improve in near future [does not think so]	0.39 (2.28)^*^	0.41 (2.41)^*^	0.38 (1.80)+	0.40 (1.90)+
Age	0.62 (4.99)^**^	0.62 (4.93)^**^	0.37 (2.67)^**^	0.36 (2.64)^**^
Age^2^	−0.01 (−6.09)^**^	−0.01 (−6.02)^**^	−0.01 (−3.89)^**^	−0.01 (−3.86)^**^
Currently married [not married]	1.77 (6.06)^**^	1.79 (6.02)^**^	0.82 (2.22)^*^	0.80 (2.15)^*^
Previously married [not previously married]	−1.05 (−3.89)^**^	−1.09 (−4.05)^**^	0.11 (0.31)	0.10 (0.30)
Number of living children	−1.07 (−10.61)^**^	−1.07 (−10.52)^**^	−0.77 (−7.88)^**^	−0.76 (−7.84)^**^
Excellent/good self-rated health [not excellent/good]	0.26 (1.18)	0.27 (1.20)	−0.04 (−0.16)	−0.04 (−0.16)
Education [basic and secondary general]				
secondary special	0.13 (0.48)	0.09 (0.34)	−0.20 (−0.76)	−0.20 (−0.76)
some tertiary or higher	0.00 (0.01)	−0.07 (−0.26)	−0.08 (−0.29)	−0.11 (−0.38)
Very religious [somewhat or not religious]	0.18 (0.54)	0.19 (0.57)	0.12 (0.40)	0.13 (0.44)
Employment status [not employed]				
employed in state sector	0.13 (0.51)	0.14 (0.57)	0.81 (2.92)^**^	0.79 (2.84)^**^
employed in private company	0.27 (0.91)	0.23 (0.78)	0.38 (1.05)	0.33 (0.90)
family business or self-employed	0.25 (1.24)	0.25 (1.20)	0.58 (1.98)+	0.58 (1.98)+
Household income per person, tier [low]				
medium	−0.28 (−1.29)	−0.31 (−1.42)	0.16 (0.70)	0.12 (0.52)
high	−0.22 (−0.83)	−0.24 (−0.91)	0.23 (0.81)	0.20 (0.69)
not known/not reported	0.20 (0.64)	0.17 (0.54)	0.68 (1.44)	0.66 (1.40)
Lives in rural area [lives in urban area]	0.23 (1.13)	0.29 (1.42)	−0.55 (−2.22)^*^	−0.50 (−1.95)+
Survey Round II [Round I]	0.75 (3.08)^**^	0.77 (3.15)^**^	0.84 (3.75)^**^	0.82 (3.71)^**^
Intercept	−6.37 (−3.11)^**^	−6.42 (−3.10)^**^	−1.76 (−0.75)	−1.81 (−0.76)
*Wants child later*				
Ethno-linguistic background				
Kyrgyz all (ref.)	0	−	0	-
Less-Russified (ref.)	-	0	-	0
More-Russified	-	0.96 (3.23)^**^	-	0.57 (1.82)+
Uzbek	−1.05 (−3.35)^**^	−0.81 (−2.55)^*^	−1.01 (−3.45)^**^	−0.84 (−2.72)^**^
European	−0.59 (−2.02)^*^	−0.17 (−0.57)	−0.55 (−1.52)	−0.30 (−0.77)
Other	−1.05 (−2.41)^*^	−0.76 (−1.75)+	0.05 (0.10)	0.26 (0.55)
All friends are of same ethnicity [not all]	0.43 (2.21)^*^	0.48 (2.42)^*^	0.27 (1.13)	0.33 (1.34)
Thinks that situation of own ethnicity will improve in near future [does not think so]	0.18 (0.99)	0.23 (1.30)	0.28 (1.33)	0.31 (1.45)
Age	0.25 (1.91)+	0.24 (1.81)+	−0.15 (−0.99)	−0.15 (−1.00)
Age^2^	−0.01 (−3.41)^**^	−0.01 (−3.32)^**^	−0.00 (−0.43)	−0.00 (−0.42)
Currently married [not married]	0.50 (1.51)	0.57 (1.73)+	−0.15 (−0.37)	−0.19 (−0.44)
Previously married [not previously married]	−0.47 (−1.76)+	−0.52 (−1.97)^*^	0.42 (1.25)	0.41 (1.22)
Number of living children	−0.46 (−4.32)^**^	−0.45 (−4.20)^**^	−0.16 (−1.56)	−0.15 (−1.47)
Excellent/good self-rated health [not excellent/good]	0.37 (1.59)	0.40 (1.69)+	0.31 (1.31)	0.30 (1.25)
Education [basic and secondary general]				
secondary special	0.50 (1.80)+	0.40 (1.42)	−0.33 (−1.24)	−0.36 (−1.37)
some tertiary or higher	0.66 (2.40)^*^	0.49 (1.79)+	0.33 (1.21)	0.26 (0.95)
Very religious [somewhat or not religious]	−0.08 (−0.23)	−0.06 (−0.17)	−0.05 (−0.16)	−0.02 (−0.06)
Employment status [not employed]				
employed in state sector	−0.21 (−0.82)	−0.18 (−0.70)	0.75 (2.31)^*^	0.73 (2.24)^*^
employed in private company	0.10 (0.35)	−0.03 (0.09)	0.71 (1.77)+	0.62 (1.55)+
family business or self-employed	−0.17 (−0.68)	−0.17 (−0.71)	0.57 (1.76)+	0.58 (1.78)+
Household income per person, tier [low]				
medium	−0.13 (−0.53)	−0.18 (−0.77)	0.08 (0.31)	0.02 (−0.08)
high	−0.09 (−0.30)	−0.19 (−0.67)	0.48 (1.57)	0.42 (1.33)
not known/not reported	0.09 (0.27)	−0.02 (−0.06)	1.05 (1.97)+	0.99 (1.84)+
Lives in rural area [lives in urban area]	−0.09 (−0.37)	0.06 (0.27)	−0.78 (−2.86)^**^	−0.67 (−2.43)^*^
Survey Round II [Round I]	0.71 (2.84)^**^	0.73 (2.95)^**^	0.48 (1.87)+	0.48 (1.86)+
Intercept	−0.32 (−0.16)	−0.52 (−0.25)	6.48 (2.61)^*^	6.32 (2.53)^*^
*N*	1597		1714	

Notes: Reference categories in brackets. Significance level:

+ p<0.10

* p<0.05

** p<0.01.
